# Supplementary material for: Molecular basis of IRGB10 oligomerization and membrane association for pathogen membrane disruption
Source: Commun Biol. 2021 Jan 19;4:92. doi: 10.1038/s42003-020-01640-7 (PMC7815755; doi:10.1038/s42003-020-01640-7)
Supplement: Supplementary file 2 — Supplementary Information [file 42003_2020_1640_MOESM2_ESM.pdf]

Molecular basis of IRGB10 oligomerization and membrane association for pathogen membrane disruption

Hyun Ji Ha[1], Hye Lin Chun[1,2], So Yeon Lee[1,2], Jae-Hee Jeong[3], Yeon-Gil Kim[3], and Hyun Ho Park[1,2,*]

[1]College of Pharmacy, Chung-Ang University, Seoul 06974, Republic of Korea.

[2]Department of Global Innovative Drugs, Graduate School of Chung-Ang University, Seoul 06974, Republic of Korea

[3]Pohang Accelerator Laboratory, Pohang University of Science and Technology, Pohang 790-784, Republic of Korea

Running title: Crystal structure of IRGB10

[*]**Corresponding authors:**

Hyun Ho Park

College of Pharmacy, Chung-Ang University

Email: xrayleox@cau.ac.kr

Phone: +82-2-820-5930

Fax: +82-53-810-4769

Supporting Information

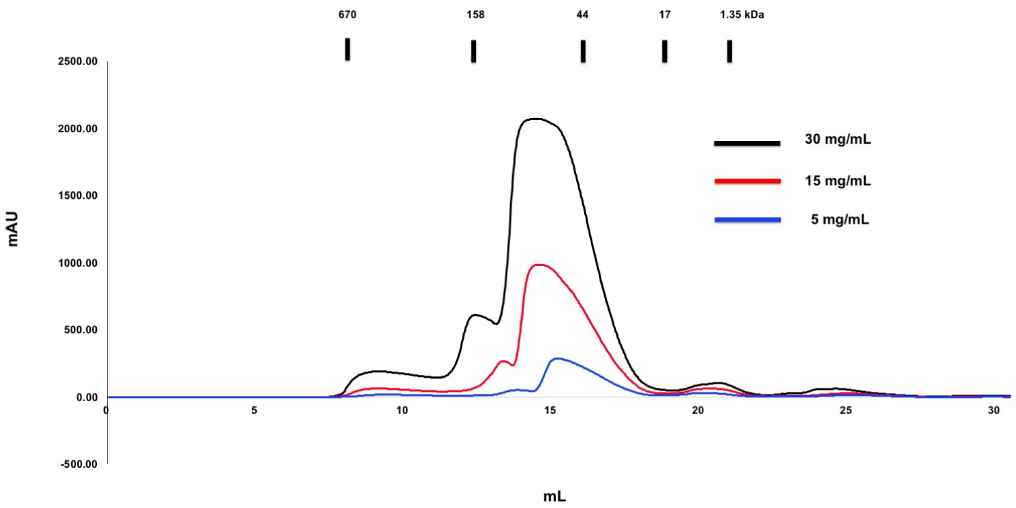

Supplementary Figure 1.
Concentration dependent oligomerization of IRGB10. SEC profile produced by various different concentrations if IRGB10.

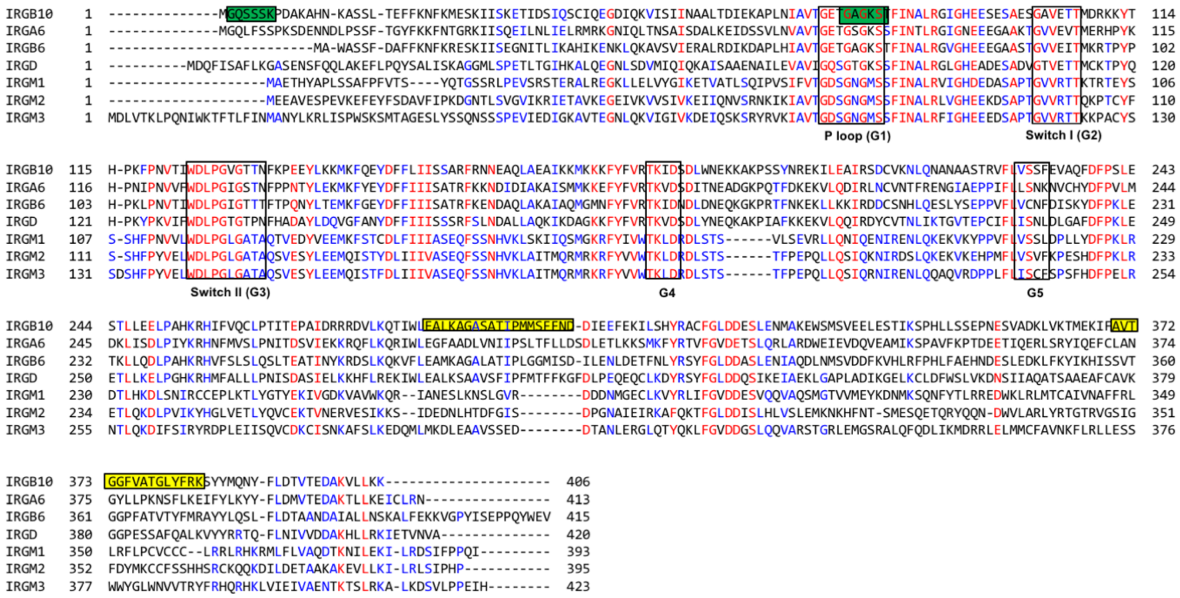

Supplementary Figure 2.
Sequence alignment of the IRG family including IRGB10. Mostly conserved and partially conserved residues are shown in red and blue color, respectively. The sequences forming five loops around the GDP binding pocket (G1~G5) which are conserved in the IRG family are delineated with black boxes. Putative myristoylation sites and putative transmembrane regions, which might be important for membrane binding, are shown by green-filled and yellow-filled boxes, respectively.

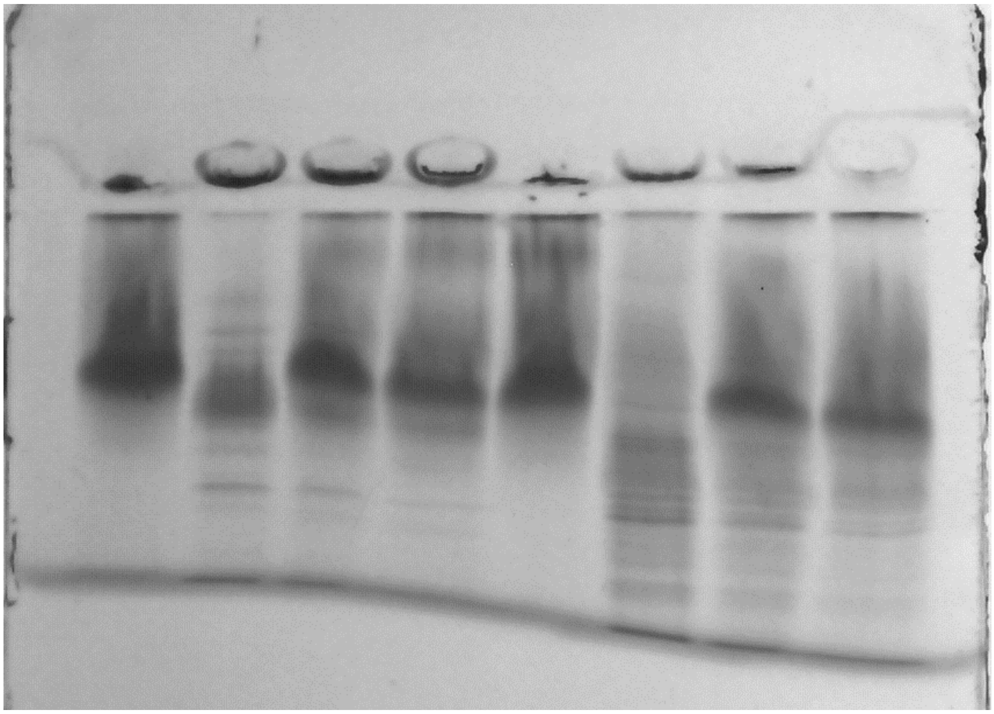

Supplementary Figure 3.
Uncropped gel used in figure 5c.

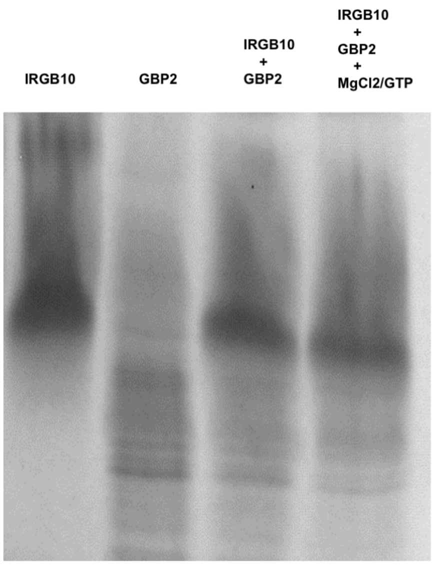

Supplementary Figure 4.
Analysis of direct interaction between IRGB10 and GBP2 by native-PAGE. 200 mM GTP and 10 mM MgCl2 was used for analyzing the effect of GTP and MgCl2.

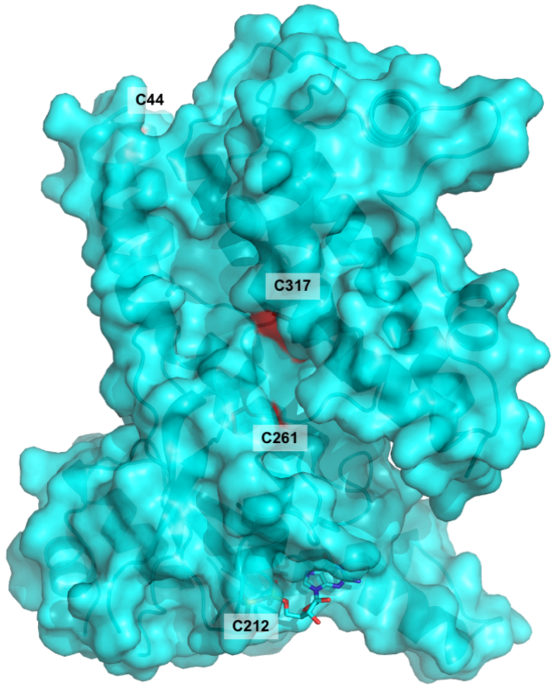

Supplementary Figure 5.
The location of four cysteines on the structure of IRGB10. Red color indicates the position of cysteines.
